# Use of Very High Spatial Resolution Commercial Satellite Imagery and Deep Learning to Automatically Map Ice-Wedge Polygons across Tundra Vegetation Types

**DOI:** 10.3390/jimaging6120137

**Published:** 2020-12-11

**Authors:** Md Abul Ehsan Bhuiyan, Chandi Witharana, Anna K. Liljedahl

**Affiliations:** 1 Department of Natural Resources and the Environment, University of Connecticut, Storrs, CT 06269, USA; Chandi.Witharana@uconn.edu; 2 Woods Hole Research Center, Falmouth, MA 02540, USA; aliljedahl@whrc.org

**Keywords:** permafrost, Arctic, deep learning, tundra, ice-wedge polygon, Mask R-CNN, satellite imagery

## Abstract

We developed a high-throughput mapping workflow, which centers on deep learning (DL) convolutional neural network (CNN) algorithms on high-performance distributed computing resources, to automatically characterize ice-wedge polygons (IWPs) from sub-meter resolution commercial satellite imagery. We applied a region-based CNN object instance segmentation algorithm, namely the Mask R-CNN, to automatically detect and classify IWPs in North Slope of Alaska. The central goal of our study was to systematically expound the DLCNN model interoperability across varying tundra types (sedge, tussock sedge, and non-tussock sedge) and image scene complexities to refine the understanding of opportunities and challenges for regional-scale mapping applications. We corroborated quantitative error statistics along with detailed visual inspections to gauge the IWP detection accuracies. We found promising model performances (detection accuracies: 89% to 96% and classification accuracies: 94% to 97%) for all candidate image scenes with varying tundra types. The mapping workflow discerned the IWPs by exhibiting low absolute mean relative error (AMRE) values (0.17–0.23). Results further suggest the importance of increasing the variability of training samples when practicing transfer-learning strategy to map IWPs across heterogeneous tundra cover types. Overall, our findings demonstrate the robust performances of IWPs mapping workflow in multiple tundra landscapes.

## 1. Introduction

Ice wedges are common permafrost subsurface features developed by repeated frost cracking and ice-vein growth over centuries to millennia [1,2,3]. These wedge-shaped ice bodies are responsible for creating polygonized land surface patterns (ice-wedge polygons, IWPs) across the Arctic [3,4]. In recent decades, abrupt thaw of ice-rich permafrost has been documented at several locations across the Arctic that alters the microtopography and the type of IWP [5].

Geographical coverage, remoteness, and logistical challenges constrain field-based mapping of IWPs. Very high spatial resolution (VHSR) commercial satellite sensors provide opportunities to observe IWPs at multiple spatial scales and temporal frequencies [6,7,8,9,10,11,12,13]. The bulk of traditional remote sensing image classification methods fail to grapple with sheer data volumes and scene complexities of VHSR imagery [14]. Increasing spectral heterogeneity in VHSR imagery leads to less class variance, which makes it difficult to accurately resolve IWPs using conventional per-pixel-based algorithms [15]. Local-scale analysis based on high-resolution data and regional-scale analysis based on coarse-resolution Landsat data limit our capacity to elucidate the effect of sub-meter scale IWP degradation on regional-scale processes, such as carbon projections [16]. Therefore, there is a need and an opportunity for advanced image analysis approaches for the accurate characterization of ice-wedge polygonal networks [16,17,18,19].

Owing to the upsurge of faster and affordable hardware resources (GPU/CPU) and easy access to cloud computing environments, deep learning (DL) algorithms are securing an increasing popularity in a wide spectrum of scientific disciplines that rely on artificial intelligence (AI). The application horizon spans from drug discovery through autonomous navigation to earth and environmental modeling [20,21,22,23,24]. Deep learning (DL)-based convolutional neural network (CNN) DLCNN have successfully outperformed other conventional machine learning techniques, such as support vector machine (SVM) and random forest (RF) in everyday image understanding. Proven success in CV has been an enticing factor for remote sensing community towards DLCNN [25,26,27,28,29]. There has been an expeditious uptake of DLCNN in VHSR image scene understanding [30,31,32,33]. Similar to everyday image analysis, DLCNN outperforms traditional machine learning classifiers (e.g., RF and SVM) as well as modern paradigms like object-based image analysis [33]. Application spectrum of DLCNN in remote sensing image to assessment pipelines is broad and multitude. It has been demonstrated that DLCNN is applicable image processing applications such as fusion [34,35], segmentation [36,37], and registration [38,39].

A growing body of studies investigated object detection, semantic segmentation, and semantic object instance segmentation using the region-based CNN (RCNN) architectures, such as Fast RCNN [40], Faster R-CNN [41], RetinaNet [42], RFCN [43], Mask R-CNN [44,45]. While object detection performs finding and classifying objects in an image, semantic image segmentation moves further steps ahead, identifying objects within a scene and labelling them according to known classes. U-Net and its successor architectures [46] are also capable of performing semantic object instance segmentation [47]. Among other comparison studies, [46] probed into two key semantic object instance segmentation architectures, U-Net and Mask-RCNN, to exploit their performances. According to their results, Mask-RCNN produced better recall and precision than U-Net, suggesting that it can detect targets of interest more accurately, although Mask-RCNN struggled to predict a good segmentation mask [46]. In considering the amount of under (over) segmentation, Mask-RCNN had much better performances compared to U-Net [46]. Specifically, in remote sensing applications Mask R-CNN was successfully applied to relatively small, selected areas for mapping of IWPs [7,10,11]. The original Mask RCNN is trained based on the COCO image data set, which harbors massive amount of hand annotated everyday images [48]. Moreover, to evaluate the automatic detection and classification of IWPs from sub-meter resolution commercial satellite imagery, transfer learning and adoption of existing Mask RCNN architecture (or pre-trained COCO data-based) could be the key solution in remote sensing applications. It is noted that our candidate algorithm, its architecture, and its underlying training data comfortably fall under the commonly found use-cases where the user is challenged by limited data and computation resources, and perhaps technical competencies. Therefore, in this study, we used Mask R-CNN for mapping IWPs in satellite remote sensing imagery to examine the transferability of model for varying tundra types such as sedge, tussock sedge, and non-tussock sedge.

Translating DLCNN from computer vision applications to the remote sensing image analysis domain undoubtedly create rich opportunities, as well as new challenges. Unlike in everyday image understanding, in which the targets in question operate in a constrained space, remote sensing imagery captures the nadir views of spatially continuous to discontinuous geo objects. Landscapes are complex. The constituent geo objects exhibit complex spectral, spatial, textural, and contextual characteristics that are aggregated across scales [49,50]. Increased resolution of commercial satellite imagery spontaneously inherit the landscape complexity. The high-level semantics that we sought for a given target could be influenced by the landscape complexity. The intriguing question is what the interoperability of a model is when it is trained in one landscape and applied to another landscape to classify the same object of interest. One could rule out this argument when the model is trained and applied in the same landscape or closer proximity. However, it is difficult to overlook this when thinking about regional scale mapping applications where we come across distinct landscapes with their own heterogeneities attributed by ecological and geophysical factors. This holds great validity in our mapping effort. We are not confined to few square kilometers but expands across whole Arctic tundra. Although semantically we pursue an abstracted geo object of polygon, its low-level motifs (spectral, spatial, textural characteristics) and high-level meanings are greatly influenced by the landscape where it stands on. Arctic tundra represents a complex and heterogeneous mosaic shaped by the earth processes, markedly influencing vegetation, hydrology, or soil characteristics [51,52]. When investigating how particular targets of interest or geo objects are presented in image modality across a region essentially, we require a baseline fabric that decomposes the heterogeneous system into meaningful patches with underlain ecological functions. In this respect, tundra cover types can stand as representative analysis units to understand how certain geo objects, for example ice-wedge polygons, present themselves in different tundra types and how their image representations change from tundra cover to another. In this respect, circumpolar Arctic vegetation map (CAVM) [53] presents a unique opportunity to use baseline data layers to aggregate ice-wedge polygons into different cohorts. Because the CAVM classification scheme not only considers vegetation types but prudently takes into account variability of topography, geomorphology, and climatic factors.

**Figure 1:**
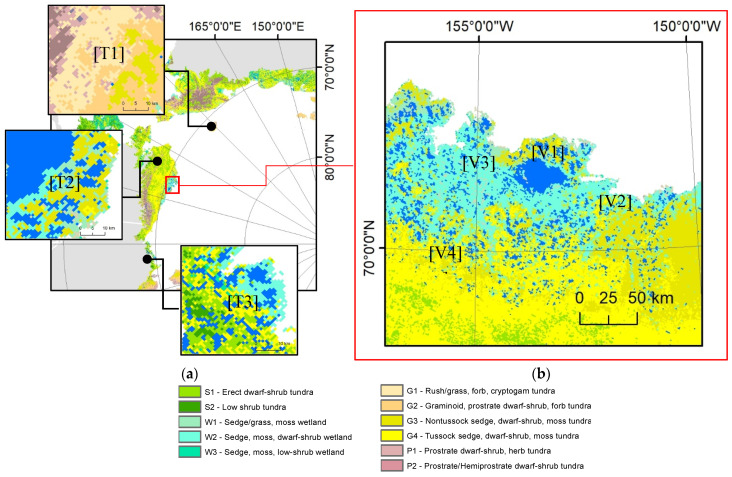
Geographical locations for training and independent validation sites: (**a**) training sites from Russia, Canada, and Alaska, and (**b**) independent validation sites from Alaska. Tundra vegetation map and the legend are adapted from [54]. Satellite imagery Copyright DigitalGlobe, Inc.

The importance of the Arctic tundra types associated with ice-wedge polygons dominates within the Circum-Arctic permafrost region [54]. The broad-scale assemblages of Arctic tundra constitute erect shrublands, graminoid tundra, mountain complexes, barrens, mineral graminoid tundra, prostrate-shrub tundra, and wetlands [55]. Figure 1 presents a snapshot of tundra types (details in [54]), which are considered in this study. Understanding of tundra distributions provides essential insight into IWP mapping application. For example, lake-rich regions such as Alaska’s North Slope demonstrated dominant sedges tundra, which contains more detailed information for IWPs mapping for that tundra type. Alaska represents heterogeneous tundra types such as tussock sedge, dwarf shrub, and moss tundra [55]. Moreover, the central portion of the Seward Peninsula, and mountain complexes concentrated in the Brooks Range of northern Alaska [54]. It is noted that ice-wedge degradation is higher in areas with warmer permafrost, like the Seward Peninsula in Alaska [56]. In addition, Russia has mostly low-shrub tundra in the Arctic, which is a consequence of predominantly wet soil moisture conditions that result from near-surface permafrost [54]. Canada has the most terrain associated with abundant barren types and prostrate dwarf-shrub tundra in in the Arctic region [54]. Therefore, it is important to consider the transient nature and spatial heterogeneity [57,58] of tundra types for the IWP mapping application.

Pilot studies have been conducted, including our efforts [59] and related work, such as [7,10,11] to demonstrate the adaptability of DLCNN in automated ice-wedge polygon detection and classification. These works exercised the transfer learning strategy by adapting one of the semantic object instance segmentation algorithm Mask R-CNN architecture that descends from the region-based CNN family. Degree to which a given DLCNN model interoperable across a heterogeneous landscapes, i.e., training and validation the model across tundra types, have been overlooked in literature. Accordingly, it is unknown how the model performs over a range of tundra cover types, such as sedge, tussock sedge, and non-tussock sedge (Figure 1b), each of which exhibit unique spectral, textural, spatial, and contextual characteristics. Prior to any regional-scale applications, the model’s invulnerability to landscape perturbations needs to be systematically quantified. These unanswered questions provide the impetus for our study. We are in the process of developing a mapping application for Arctic permafrost land environments, which enables the transformation of large volumes of commercial satellite imagery into Arctic science ready applications. Our main goal of the current study is to explore the DLCNN model interoperability across different tundra types and image scene complexities in order to understand the opportunities and challenges prior to any future circumpolar IWPs mapping applications. Migration of landscape complexities to image scenes evidently pose new challenges on automated image processing using DLCNN model predictions. Our experimental design aim to encapsulate low-gradient Arctic upland tundra (sedge, tussock sedge, and non-tussock sedge), including various features such as lakes and vegetated drained thaw lake basins. We aim to (1) examine the transferability of the model in mapping IWPs across tundra types; (2) evaluate the automatic detection and classification of ice-wedge polygons from sub-meter resolution commercial satellite imagery.

## 2. Study Area and Data

We conducted our study based on four summer-time multi-spectral images acquired by the WorldView-2 satellite sensor (Figure 1b). Pansharpened multispectral images at 0.5 m were provided by the Polar Geospatial Center as orthorectified, atmospherically corrected data products. Four candidate image scenes and their respective features are presented in Table 1. Candidate scenes cover 1500 km^2^ of coastal and upland tundra from the North Slope, Alaska (Figure 1b). The training datasets, which were represented by different image scenes than the evaluation assessment, were established around different tundra covers and included imagery from Alaska, Canada, and Russia (Figure 1a). Table 2 presents different tundra types for training and validation sites. Spectral characteristics significantly vary across the different tundra types [60]. The training sites provide a substantial landscape heterogeneity for model classifying and detection IWPS. Moreover, dominant landcover types (heterogeneity) controls the global image statistics [61]. Therefore, choosing image scenes from varying tundra could greatly influence model training since the model earns the opportunity to learn different abstractions of the targets of interest.

## 3. Mapping Application for Permafrost Land Environment

Accurate characterization of IWPs from VHSR imagery directly depend on the segmentation (i.e., isolation of targets from the surrounding) and classification (i.e., assigning the correct label to the targets) processes [62,63]. Semantic object instance segmentation methods are designed to afford target isolation and labeling to thematic classes. Ideally, a mapping application for permafrost land environment should consist of candidate DLCNN models tailored to extract different permafrost features of interest from remote sensing imagery. Among the suite of target features, microtopography, thaw features, capillaries, and plant functionality exhibit high priority. Given the diversity of target features and their heterogeneous characteristics coupled with semantic complexities, multiple model architectures better serve the purpose. In our mapping application, one pipeline targets on mapping ice-wedge polygons in which we used Mask RCNN algorithm. The pipeline is extensible and tailored to work with remote sensing imagery using high-performance computing resources. This allows scalability to larger spatial extents.

### 3.1. Mapping Workflow, Training and Validation Experiment

**Figure 2:**
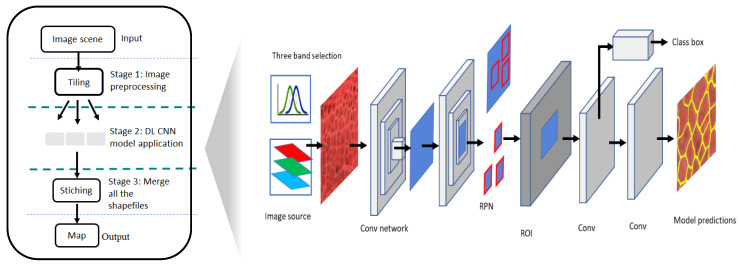
Simplified schematic of the automated ice-wedge polygon mapping workflow (**left**) and the general architecture of the Mask R-CNN algorithm (**right**).

We center the current mapping workflow using Mask R-CNN, which uses the multi-level features from the training sample for detection, delineation, and classification of targets of interest. Similar to the other member of RCNN family, in a generic sense, MRCNN is a two-stage detector. Its architecture comprises of sub-networks: (1) generates Region Proposal Network (RPN) (i.e., candidate object bounding boxes); and (2) predicts the class, bounding box, and binary mask for each region of interest (ROI). The Mask R-CNN uses Residual Learning network, ResNet (101 layers deep), a convolutional neural network [44] for feature extraction. The pretrained network can classify images into multiple object categories which helps to converging deeper networks. In the deeper network the additional layers better approximate the mapping which reduces the error by a significant margin. Our workflow is modular. It consists of several key stages as depicted in Figure 2. In stage 1, the main input to the workflow is multispectral satellite imagery with three bands, with radiometric depth of 16 bit. Image scenes from the Polar Geospatial Center are typically provided with the dimension of 20 km (40,000 pixel) × 20 km (40,000 pixel) at 0.5 m pixel resolution. To achieve the optimal combination of spectral bands from input multispectral imagery which contain more than three bands (for instance, WV02 imagery has 8 spectral bands: coastal blue, blue, green, yellow, red, coastal red, NIR1 and NIR2), we used three statistical measure: variances, probability distribution function (PDF), and cumulative distribution function (CDF) (details in [59]). Specifically, a systematic experiment was designed to understand the impact of choosing the optimal three-band combinations in the use of multispectral datasets on DLCNN model prediction [59]. As the first step in the pipeline, the most effective combination of bands is obtained by estimating variances where the best three channels approximately present similar spread [59,64,65]. As three bands produce approximately similar reflectance values from PDF, we consider those three bands for the proposed model [66,67,68]. We also examined the shape of the cumulative density function (CDF) and observed the magnitude of multispectral bands [59,69]. CDF explains the distribution of the reflectance values among multiple spectral bands and, for the workflow, we chose the considerably less deviated three bands. Finally, for each spectral band of the image scene, the best combination of three bands was obtained by estimating three statistical measures: variances, PDF and CDF. In stage-2, the input image scene was portioned into tiles of 200 × 200 pixels. A typical satellite image scene produces ~65,000 tiles (this depends on the input scene size). Tiles are then streamed to the trained model for inferencing. The model estimates detection (IWPs prediction) when input tile contains ice-wedge polygons. The predicted categorical raster is vectorized as a shapefile. In stage-3, all the individual shapefiles (corresponding to each tile) will be post-processed by omitting duplicates along tile borders and merged together to create a single shapefile corresponding to the extent of the input satellite image scene.

For model training purposes, we created annotated data (defining and labelling regions of interest) using an online web tool “VGG Image Annotator” from satellite imagery comprising heterogeneous tundra types. We randomly selected 262 cropped subsets (tiles of 200 pixels by 200 pixels) (~15,000 polygons) from different tundra types (tussock, non-tussock, and sedge) considering the spectral, and spatial variability. Each file had 200 × 200 pixels. Datasets were annotated for two classes: Low-centered (LC) polygons (8962 objects) and high-centered (HC) polygons (6038 objects). It is also notable that IWPs were delineated along their edges (i.e., if troughs are present, then along the trough-sides, if no troughs are present, then along the rim mid-line). Finally, the annotated tiles were randomly divided into a training dataset, validation dataset, and test dataset based on the 8:1:1 split rule. We trained the DLCNN with a mini-batch size of two image tiles, 350 steps per epoch, learning rate of 0.001, learning momentum of 0.9, and weight decay of 0.0001 [7,12,13,59]. After scanning the image, the Mask R-CNN generates Region Proposal Network (RPN), and subsequently, the DLCNN predicted the class, bounding box, and binary mask for each region of interest (ROI) to obtain our mask prediction (the predicted mask is pixel-based). For each ROI, segmentation mask was predicted using a small Faster R-CNN. Finally, Mask R-CNN resized the predicted mask back to the original dimensions of input image scene. Training was implemented using NVIDIA V100 GPUs (PSC–Pittsburgh Supercomputing Center, Pittsburgh, PA, USA) on XSEDE supercomputing resources. We trained the DLCNN with 100 epochs. To optimize Mask R-CNN, we examined different losses such as (a) Smooth-L1 loss, defines box regression on object detection systems, which is less sensitive to outliers, than other regression loss; (b) Mask R-CNN bounding box loss indicates the difference between predicted bounding box correction and true bounding box; (c) Mask R-CNN classifier loss estimates difference of class labels between prediction and ground truth; (d) mask binary cross-entropy loss measures (probability value between 0 and 1) the performance of a classification model by observing predicted class and actual class; (e) RPN bounding box loss identifies the regression loss of bounding boxes only when there is object; and (f) RPN anchor classifier loss indicates the difference between the predicted (RPN) and actual (closest ground truth box to the anchor box) regression.

### 3.2. Accuracy Estimates

To evaluate the DLCNN performances, various error metrics were performed in the validation experiment. The mean intersection over union (mIoU) (in %) between predicted and ground truth is presented below:

(1) $$\mathrm{mIoU}=\frac{A_{O}}{A_{U}}$$

Here, $A_{O}$ indicates the area of overlap between the predicted segmentation and the ground truth, where $A_{U}\;$ is the area of union between the predicted segmentation and the ground truth. A mIoU score > 0.5 is considered a “good” prediction which indicates successful delineation [45,70].

Absolute mean relative error (AMRE) is the mean of the relative percentage error, calculated by the normalized average:

(2) $$\mathrm{AMRE}=\frac{1}{n}\overset{n}{\underset{i=1}{\sum }}|\left(\frac{{\hat{y}}_{i}-y_{i}}{y_{i}}\right)|$$

For the quantitative assessments, for each subset the number of predicted polygons $\hat{y}$, the number of actual polygons (ground-truth polygons) $y_{i}$ and n is the number of subsets (details in [71,72]).

An accurate prediction of ice-wedge polygon is represented by F1 score, where a score of 1 specifies perfect prediction. Correctness signifies how many of predicted positives were truly positive; completeness determines what percentage of actual positives were detected. An accurate prediction is represented by all metric values closing to 1. The Statistical measures used in the study are shown below.

(3) $$\mathrm{Correctness}=\frac{\mathrm{TP}}{\mathrm{TP}+\mathrm{FP}}$$

(4) $$\mathrm{Completeness}=\frac{\mathrm{TP}}{\mathrm{TP}+\mathrm{FN}}$$

(5) $$F1\;\mathrm{Score}=\frac{2*\mathrm{Correctness}*\mathrm{Completeness}}{\mathrm{Completeness}+\mathrm{Completeness}}$$

Please note that true positive (TP) is the number of polygons correctly identified, false positive (FP) is the number of polygons identified by model, but not true, and false negative (FN) is undetected polygons.

## 4. Model Evaluation Results and Discussions

**Figure 3:**
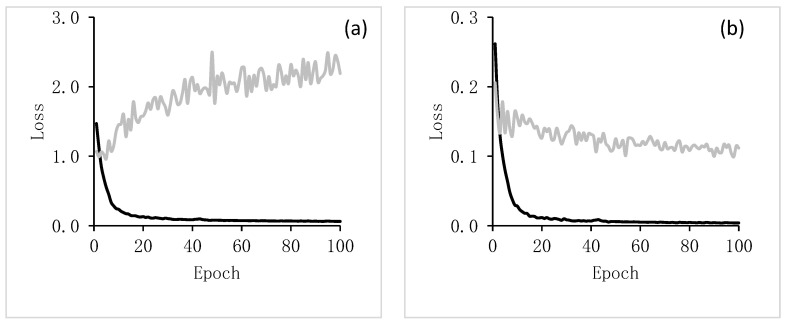

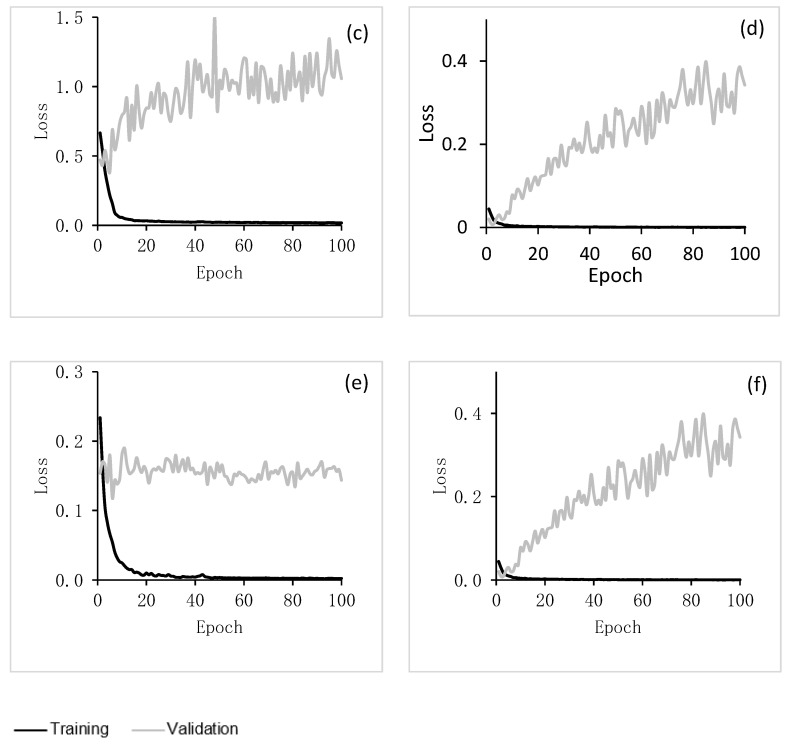
Training and validation loss of Mask R-CNN model (**a**) Smooth-L1 loss; (**b**) Mask R-CNN bounding box loss; (**c**) Mask R-CNN classifier loss; (**d**) Mask binary cross-entropy loss; (**e**) RPN bounding box loss; (**f**) RPN classifier loss.

We optimized the DLCNN during the training process with 100 epochs to get the full learning curve of the model. Learning curves are widely used diagnostic tool in machine learning for algorithms that learn from a training dataset incrementally. Overall model learning performance over experience or time are presented by a learning curve as shown in Figure 3. Results show the changes in learning performance for different epochs over time, where an epoch is defined as the number of times an algorithm visits the data set (e.g., an epoch is one backward and one forward pass for all the training). The validation loss values reached their lowest at 2nd epoch (Figure 3). Therefore, we choose the Mask R-CNN model with the lowest validation loss for our experiments (i.e., the 2nd epoch). It is noted that the sample sizes are limited but sufficient to optimize the model for limited number of epochs (2nd). Specifically, from the results of the Smooth-L1 loss (target detection loss), the validation loss values reached their lowest magnitude at 2nd epoch, but the training loss values substantially decreased (Figure 3a). Similarly, Figure 3b–f, in considering other losses (Mask R-CNN bounding box loss; Mask R-CNN classifier loss; Mask binary cross-entropy loss; RPN bounding box loss; RPN classifier loss), showed that around the 2nd epoch, the validation loss value reached its lowest value, where Mask R-CNN was optimized. In our use case, we practiced transfer learning of existing Mask RCNN architecture to optimize the model at low number (e.g., 2nd) of epoch to evaluate the automatic detection and classification of ice-wedge polygons from sub-meter resolution commercial satellite imagery.

We statistically evaluated the performances of the DLCNN in detecting and classifying IWPs. For the quantitative assessments, from each image scene, we randomly selected 40 subsets to manually delineate polygons as a reference (ground-truth polygons). The mIoU values varied between 0.85 to 0.91 (Table 3), which indicted that predicted polygons that agree with the ground-truth polygons.

**Figure 4:**
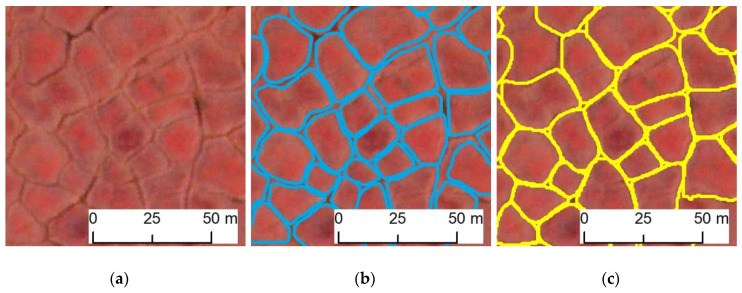
Zoomed-in views of (**a**) original imagery, (**b**) ground truth (manual delineation, blue outline) and (**c**) model result (yellow outline) for candidate scene V4. Imagery © [2016] DigitalGlobe, Inc.

**Figure 5:**
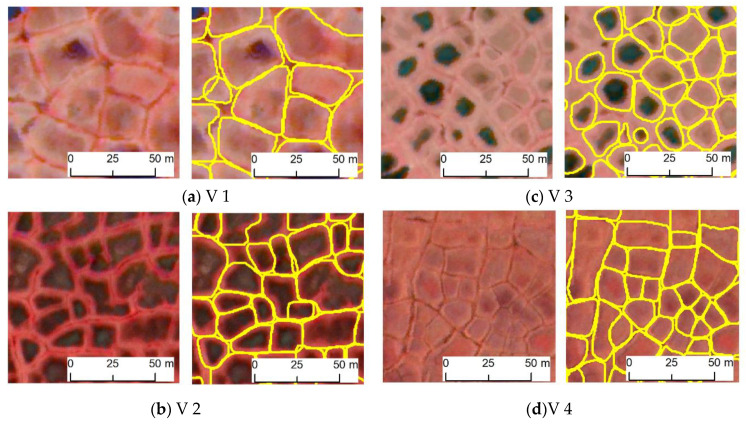
Sample views of original imagery (left) and model classification (right) for candidate scenes. Yellow outlines denote automatically detected IWPs. Imagery © [2010, 2012, 2015, 2016] DigitalGlobe, Inc. Satellite image scenes are obtained from different tundra regions: (**a**) non-tussock sedge tundra; (**b**) non-tussock sedge tundra; (**c**) sedge tundra; (**d**) tussock sedge tundra.

Close-up views of the original imagery, ground truth, and model classification results show that our predicted IWPs closely matched ground-truth IWPs (Figure 4). We used three quantitative error statistics (correctness, completeness, and F1 score) to show the performances of the framework (Table 4). Candidate scenes V1, V2, V3, and V4 produced high model detection accuracies for the F1 score, ranging from 0.89 to 0.96 (Figure 5, Table 4). Although all the image scenes are geographically close to each other, they still have different tundra variations in the microtopography. Predominance of tussock sedge tundra and the high spatial resolution of imagery information provide landscape-scale variation within the original CAVM map throughout northern Alaska [54,55]. Scene V4, covering tussock-sedge achieves mIoU 0.85 (Table 3), still having a chance to improve model prediction by increasing more training data from that tundra region. Specifically, V1, V2, and V3 represents non-tussock sedge and sedge tundra types of Alaska’s North Slope. DLCNN performances for Image scenes V2–V3 (F1 score: 0.92–0.94) were consistent, which means the training samples were sufficient to predict IWPs for non-tussock sedge and sedge tundra regions (Table 4). This result helped to understand the feasibility and reliability of the remote sensing information extraction for different tundra regions. In terms of elevation, most of the Arctic is <500 m elevation, with the lowest elevations (<100 m) dominated by graminoid-erect dwarf-shrub tundra. In our training datasets, we used three tundra types where elevation varies up to 500 m. Our validation image scenes V(1–4) are found within this elevation range and exhibited relatively consistent performances across the selected validated tundra regions. In future, more training sample from the complex terrain with the highest elevations could increase the greatest variability of the model.

In a similar fashion, scenes V1-4 scored high values for completeness (81–89%). In all four cases, the correctness metric scored 1, allowing less freedom for false alarms. There are few recent studies considering 0.5 × 0.5 m resolution image where F1 scores were 55% [10], and 72% [11], which are substantially lower than the results presented here (>89%). Moreover, classification accuracies for the F1 score varied from 0.94 to 0.97 for candidate scenes, indicating a robust performance of the DLCNN algorithm across different tundra types in northern Alaska (Table 5). Remote sensing image data with more than three bands have not yet been able to be trained in deep learning training networks. Specifically, deep learning (DL)-based past researchers are designed to accept standard RGB bands as they confront with everyday images [73,74,75]. Moreover, in terms of using multispectral perspective, the Arctic tundra vegetation communities have separable view in Arctic mapping application [76,77]. The tundra types such as wet sedge meadow, tussock tundra, etc., showed certain diagnostic reflectance which were significantly different for the other tundra types [76]. On the other hand, our mapping workflow optimized multispectral band combination from satellite imagery [59], which led to a more robust image classification model than a traditional object-detection model. Moreover, results showed significantly low systematic errors (AMRE values from 0.17 to 0.23) for all candidate scenes (Table 6). Overall, both quantitative and qualitative evaluations support the possible interoperability of the IWPs mapping algorithm across different tundra assemblages in northern Alaska.

In this exploratory study, we primarily investigated the interoperability of deep learning model predictions across heterogeneous tundra landscapes. Arctic tundra vegetation exhibits a significantly higher degree of heterogeneity over small spatial scales [52]. Further research is needed to better understand how trained models behave across other tundra vegetation types and regions. Such study would also benefit from incorporating terrain units, soil types, hydro-climatic regimes, and permafrost characteristics. Furthermore, summer temperature variations can cause major changes to vegetation structure via by pose spectral/textural changes in the acquired imagery. Thus, the seasonality could be an important factor deciding the appearance of ice wedge polygon on the satellite imagery because changes to spectral and textural characteristics can alter the overall semantics of the target. The model can therefore be biased if it is only trained on imagery acquired in a particular time window. Operator biasness in hand-annotated data production can also negatively influence model performances. Tasking multiple operators to produce sizeable amount of quality-controlled training datasets can help improving the variability training samples and eventually leveraging the model performances. In future research, we aim to systematically probe further into model interoperability considering multi-faceted factors. Moreover, Arctic tundra landscapes cover spatially isolated ponds, lakes, marshes, river, and stream corridor wetlands, which representing highly heterogeneous features, varying in soil moisture, vegetation composition, elevation, surficial geology, ground ice content, soil thermal regimes and surface hydrology [51,52]. Fine-scale differences in microtopography, limit the ability to comprehend local scale controls on regional to global scale patterns which, is an important factor in characterizing IWPs in Arctic varying tundra areas [62].

## 5. Conclusions

Here we presented a deep learning CNN-based high-performance mapping application for permafrost environments to automatically characterize ice-wedge polygons from VHSR commercial satellite imagery across three common tundra vegetation types. The DL model exhibited promising performances (high detection accuracies: 89% to 96% and high classification accuracies: 94% to 97%) across the heterogeneous tundra regions. Consideration of contextual information (e.g., edges, vegetation, shape, area, and the consistency of feature distributions) increased the reliability of the model classification and helped generalizing the DL model across tundra vegetation types. Complex topography plays a vital role in controlling the spatial variation in image scenes. In this exploratory study, we used varying tundra types (sedge, tussock sedge, and non-tussock sedge) and image scene complexities to refine the understanding of opportunities and challenges for regional-scale mapping applications. However, Arctic tundra includes additional vegetation types. Therefore, the model can be biased when it is applied to other tundra vegetation types such as prostrate dwarf-shrub, herb, lichen tundra; rush/grass, forb, cryptogam tundra; graminoid, prostrate dwarf-shrub, forb tundra, etc. In the future, this experiment can be extended considering more diverse tundra landscapes, such as graminoid and shrub dominated vegetation cover types, to systemically gauge the improvement of the DL model prediction accuracies.

Effort to further refine model prediction accuracies could include (a) increasing the variability of training samples with additional annotated IWPs from a larger set of tundra vegetation types, and (b) exploring more sophisticated image pre-processing steps such as differing data fusion (pansharpening) approaches. Such model improvements may be able to produce more pronounced IWPs edge information and, therefore, improving the DL model prediction accuracies.

## Figures and Tables

**Table 1:** General characteristics of four candidate image scenes.

Site	Sensor	Acquisition Date	Image Off Nadir	Sun Elevation	Azimuth
V1	WorldView2	07/29/2010	38.6°	35.8°	135.5°
V2	WorldView2	07/04/2012	27.2°	42.2°	47.6°
V3	WorldView2	07/05/2015	15.4°	42.4°	203.8°
V4	WorldView2	09/03/2016	25.9°	27.8°	207.6°

**Table 2:** Different tundra types for training and validation sites.

Dataset	Study Sites		Dominant Tundra
Training	Russia	T1	G1-Rush/grass, forb, cryptogam tundra G2-Graminoid, prostrate dwarf-shrub, forb tundra P1: Prostrate dwarf-shrub, herb, lichen tundra P2: Prostrate/Hemiprostrate dwarf-shrub
	Alaska	T2	G4 Tussock-sedge, dwarf-shrub, moss tundra
	Canada	T3	G4:Tussock-sedge, dwarf-shrub, moss tundra G3:Non-tussock sedge, dwarf-shrub, moss tundra W2:Sedge-wetland complexes
Validation	Alaska	V1	G3:Non-tussock sedge, dwarf-shrub, moss tundra W2:Sedge-wetland complexes
		V2	G3:Non-tussock sedge, dwarf-shrub, moss tundra W2:Sedge-wetland complexes
		V3	W2:Sedge-wetland complexes
		V4	G4:Tussock-sedge, dwarf-shrub, moss tundra

**Table 3:** Summary statistics of mean intersection over union (mIoU) for candidate image scenes.

Validation Sites	mIoU
V1	0.91
V2	0.87
V3	0.86
V4	0.85

**Table 4:** Accuracy assessment of detection for candidate image scenes.

Validation Sites	Number of Reference Polygons	Correctness	Completeness	F1 Score
V1	582	0.99	89%	0.96
V2	567	1	85%	0.94
V3	579	1	83%	0.92
V4	573	1	81%	0.89

**Table 5:** Accuracy assessment of classification for candidate image scenes.

Validation Sites	Number of Reference Polygons	Correctness	Completeness	F1 Score
V1	582	0.98	99%	0.97
V2	567	0.99	96%	0.95
V3	579	0.98	97%	0.96
V4	573	0.99	95%	0.94

**Table 6:** Absolute mean relative error (AMRE ) for candidate scenes.

Validation Sites	AMRE
V1	0.17
V2	0.18
V3	0.21
V4	0.23
